# A qualitative investigation of the roles and perspectives of older patients with advanced cancer and their family caregivers in managing pain in the home

**DOI:** 10.1186/1472-684X-13-39

**Published:** 2014-08-08

**Authors:** Christine J McPherson, Thomas Hadjistavropoulos, Alana Devereaux, Michelle M Lobchuk

**Affiliations:** 1 School of Nursing, Faculty of Health Sciences, University of Ottawa, 451, Smyth Road, Ottawa, Ontario K1H 8M5, Canada; 2 Department of Psychology, University of Regina, Regina, Saskatchewan S4S 0A2, Canada; 3 Faculty of Nursing, University of Manitoba, Winnipeg, Manitoba R3T 2N2, Canada

**Keywords:** Cancer, Pain, Palliative care, Family caregivers, Aging

## Abstract

**Background:**

Pain in advanced cancer is complex and multifaceted. In older patients comorbidities and age-related functional decline add to the difficulties in managing cancer pain. The current emphasis on care in the community, and preference by patients with life-limiting disease to receive care in the home, has meant that patients and their family caregivers have become increasingly responsible for the day-to-day management of cancer pain. An appreciation of patients’ and caregivers’ roles and perspectives managing pain is, therefore, fundamental to addressing cancer pain in this setting. Consequently, we sought to explore and describe their perspectives and roles.

**Methods:**

A qualitative descriptive approach was used. Semi-structured interviews were conducted with a purposeful sample of patient- family caregiver dyads. Participants included 18 patients aged 65 years and over, with advanced cancer, receiving palliative care at home, and 15 family caregivers. The interview data were analysed using thematic analyses. Strategies were used to establish rigour.

**Results:**

Two main themes were identified. The first theme, "Communicating the pain", represented pain assessment and incorporated four subthemes in which participants described: their roles in pain assessment, the identification and expression of pain, and the communication of pain between patients and caregivers. The second theme, "Finding a solution", comprised of four subthemes that reflected participants’ roles and approaches in controlling pain; as well as their beliefs about cancer pain control, experience with side effects, and perspectives on the goals of treatment.

**Conclusions:**

The findings support other studies in identifying knowledge and attitudinal barriers to pain control; while adding to the literature by highlighting practical and relational barriers faced by older patients and their family caregivers. Health care professionals can do much to address the barriers identified by: correcting misconceptions regarding cancer pain, facilitating the communication of pain within dyads, and ensuring that patients and family caregivers have the knowledge, skills, and ability to assess and implement pain treatment strategies. This support needs to be individually tailored to meet the ongoing needs of both members of the dyad so that the shared goals of pain management are accomplished.

## Background

Pain continues to be a significant issue for people with advanced cancer. Estimates across studies suggest a pooled prevalence of 64% [1] and 70% [2] with almost half of patients reporting moderate and severe levels of pain [1]. Despite advances in the treatment of cancer pain, and guidelines to optimize assessment and management [3,4], pain continues to be inadequately assessed and treated [5]. The disproportionate prevalence of cancer in old age means that many patients are likely to be older [6]. Similar to other types of pain, cancer pain in older patients tends to be under-recognised and treated [7,8]. Co-morbidity more common in old age, and age-related declines in functioning and sensory impairments, contribute toward making pain management especially challenging in this population [9,10]. At the same time, the current emphasis on care in the community, and preference by patients with life-limiting disease to receive care in the home [11], has meant home is increasingly the setting for cancer pain management. In this context although pain management is overseen by health care professionals, it is patients and their family caregivers (hereafter caregiver) who are primarily responsible for the day-to-day management of cancer pain [12-14]. Understanding patients’ and caregivers’ roles and experiences managing cancer pain is, therefore, fundamental to addressing the pain needs of older patients with advanced cancer in this setting, and for identifying barriers and challenges to effective pain control.

Cancer pain management in advanced disease is complex. Nociceptive and neuropathic pain typically occurs as a result of damage to underlying tissue and nerves; therefore, the sensory characteristics vary depending on the etiology and extent of damage [15]. As a result, it is not uncommon for patients with cancer to experience several types of pain that may require different approaches to control the pain [16]. Further, cancer pain may fluctuate over time with progressive disease and treatments, requiring ongoing modifications to pain treatment strategies. Compounding pain and its management in older patients, is the increased likelihood of comorbidities and age-related functional and sensory impairments, which can hamper cancer pain assessment and treatment [9,10], and augment the experience of pain [17]. Non-cancer chronic pain due to co-morbidities [18] also makes the possibility of both cancer and non-cancer pain more probable in this population.

Adding to the complexity is the subjective and multidimensional nature of pain [19]. Evidence supports the physiological, sensory, affective, cognitive, and behavioral components of cancer pain that collectively influence its perception, experience and management [20]. A consistent finding in the literature is the association between psychological distress and cancer pain intensity [21]. The interplay among physiological/sensory, cognitive, affective, and behavior components of cancer pain are also evident. Identifying cancer as the source of pain, for example, is associated with greater interference with activities and enjoyment of life [22], while the existential significance of pain as a sign of progressive disease and impending death [23] can intensify the experience of pain leading to suffering [24]. Given the variable characteristics of pain, and multidimensionality of the pain experience, it is not surprising to discover that patients and caregivers find managing cancer pain one of the most challenging aspects of care [12,13].

Barriers to optimal pain control at the patient and caregiver level largely reflect these intricacies. The subjective nature of pain, for instance, means there is a reliance on patients’ communication of pain for accurate and comprehensive assessment. Barriers to perception and communication of pain include: sensory and cognitive impairment [25], misconceptions regarding the inevitability of pain with cancer, and the fatalistic meaning of pain as a sign of impending death [23], concerns about burdening family members [26,27], and apprehension about distracting health care professionals [28]. The erroneous belief that pain is expected in old age is another potential barrier for older patients [25]. Evidence indicates that caregivers may share these beliefs and concerns [29]. This finding is significant because caregivers are an important support for patients managing cancer pain at home, and their views and beliefs may influence patients’ attitudes towards pain [14]. Further, as cancer progresses and patients’ functioning deteriorates, caregivers are likely to take on greater responsibilities with respect to pain management and may become a proxy for patients in interactions with health care professionals. Caregivers’ knowledge and beliefs regarding cancer pain are, therefore, likely to influence not only how they respond to patients’ pain, but also how health care professionals respond.

Effective pain management depends not only on an accurate assessment of pain, but the selection and implementation of the most effective approach taking into consideration access to pain treatment options and patients’ preferences. Research examining how cancer pain is managed in the home has highlighted the extensive roles and demands placed on patients and their caregivers [12,30-33]. A consistent finding is that caregivers’ lack the knowledge and skills to select and implement pain treatment strategies [30]. As a consequence, many patients and caregivers feel they do not have control over cancer pain [34]. Qualitative research has increased awareness of some of the difficulties encountered by patients and caregivers as they engage in the process of pain management [23,31-35]. In a qualitative study of caregivers of patients with advanced cancer, Mehta et al. concluded that the inability of caregivers to distinguish between different types of pain and to determine the most appropriate pain treatment strategy, potentially contributed to suboptimal pain management [31,33]. Studies that have examined both patients’ and caregivers’ perspectives on pain management in advanced cancer, draw attention to the complexity of managing cancer pain in the context of life-limiting disease [23,32,34,35]. The ability of patients and caregivers to problem solve with new or increasing pain, tailor prescribed regimens to the unique needs and preferences of the patient, whilst managing side effects from pain treatments, are identified as particularly difficult [32].

Even when the most effective pain treatment option is identified and available, it may not be implemented. A major barrier contributing to inadequate pain control is patients’ reluctance to take opioids [28,29,36]; a mainstay of cancer pain treatment [3]. This reticence stems from patients’ misconceptions regarding addiction and tolerance, and concerns about side effects [28,29,36]. Comparisons between younger and older patients with cancer show that these beliefs and concerns are a greater barrier for older patients [37]. Misconceptions regarding the inevitability of pain further contribute to patients’ reluctance to use pharmacological approaches [28,36]. Similarly, caregivers’ reluctance to administer medications and under medication of patients, may stem from their own misconceptions regarding tolerance, addiction, and the inevitability of pain with cancer [38,39].

Taken together, the findings emphasise the need to fully understand the perspectives of those responsible for the day-to-day management of cancer pain. Though studies have identified barriers and raised awareness of issues faced by patients with advanced cancer and their caregivers, there is relatively little understanding of how pain is managed from the dyadic perspectives of older patients and their caregivers within the home setting. The higher prevalence of cancer in old age [6], increased risk for under-treatment of pain [7,8], and complexity associated with pain management in this population [9,10], calls attention to the need to identify and address the unique needs of these patients and their caregivers. Consequently, the purpose of this study was to describe the roles and perceptions of older patients with advanced cancer and their caregivers in managing pain in the home setting. The study is part of a larger qualitative investigation examining the cancer pain experience (i.e. meaning and the shared experience of living with cancer pain) and its management from the perspectives of older patients with advanced cancer and their family caregivers. The richness and depth of participants’ responses meant that we were not able to provide depth in discussing pain perception and management within the same manuscript. Therefore, the findings regarding pain perception are presented in a related publication [40].

## Methods

A qualitative descriptive approach with an inductive thematic analysis was used to describe and interpret data obtained through semi-structured interviews with participants [41]. A naturalistic perspective was chosen as it permits comprehensive exploration of the phenomenon within its context, while recognising its complexity; necessary for fully understanding pain management within the broader context of the dyadic caregiving relationship, advanced disease and aging.

### Sample and setting

Following ethics approval from the University of Ottawa research ethics committee, a purposeful sample of patients and family caregivers was recruited through an organization that coordinates home care services across a large urban area. To be eligible, patient participants had to be: diagnosed with advanced cancer (stage III or IV); aged 65 years or older; experiencing cancer pain for at least one month; determined as cognitively able and well enough to provide consent and reliable information by the case manager identifying the patient; English or French speaking; receiving palliative care services at home. Eligible caregivers were identified by a participating patient as the person providing the majority of care at home, and were English or French speaking. Sample size was based on informational redundancy; where no new information is forthcoming [42].

To fully describe the sample we collected information on patients’ level of physical functioning with the Palliative Performance Scale (PPS) [43], and current pain intensity rating using the pain scale from the Edmonton Symptom Assessment Scale (ESAS) [44]. Both measures are widely used in palliative care and were routinely collected by the organization where recruitment occurred. The PPS includes factors that indicate physical decline in terminal illness: ambulation; activity and evidence of disease; self-care, intake, and conscious level. Categories on the PPS, range from fully ambulatory and healthy (100%) to death (0%) in 10% increments of decline [43]. The ESAS consists of ten scales used to assess nine symptoms commonly experienced in cancer; plus an optional blank item to add another symptom. Each scale comprises of 11 points. The pain scale ranges from 0 (no pain) to 10 (worst possible pain) [44].

Characteristics of patients and caregivers are shown in Table 1. The sample comprised of 18 patients and 15 family caregivers. Three caregivers were not interviewed. Of these, two caregivers could not be contacted, and in another case the patient did not want her caregiver approached. Typically patients lived with their caregivers (n = 13) and were predominantly cared for by partner caregivers (n = 11), who were themselves older. Although patients’ physical functioning varied (PPS range 40% - 90%), most required assistance with day-to-day activities (PPS median 60%). Current pain ratings using the ESAS indicated that while the range was divergent (ESAS 0-10 units), the median ESAS rating of four signified that most had moderate pain [45]. All patients were receiving palliative care through an organization that coordinates home care services and were receiving nursing care at least once every other week. Some patients were also receiving specialist pain services as outpatients, following referral from their physicians.

**Table 1:** Characteristics of patients and family caregivers

**Characteristic**	**Patient (*n*** **= 18)**	**Caregiver (*n*** **= 15)**
	***Mean (S.D.)***	***Mean (S.D.)***
Age *(years)*	77.7 (8.8)	69.9 (14.7)
	*Median (range)*	
Palliative performance scale		60 (40%-90%)	
Edmonton symptom assessment scale (pain)	4 (0-10)		
Gender	*n*	*n*	
Male	8	4	
Female	10	11	
Cultural/ethnic background *(self-identified)*			
Canadian	11	9	
French Canadian	3	3	
European	3	2	
Haitian	1	1	
Relationship between patient and caregiver			
Partner		11	
Parent/adult child		3	
Sibling		1	
Lives with caregiver			
Yes	13		
No	5		
Primary cancer site			
Breast	5		
Genitourinary	4		
Digestive/Gastrointestinal	4		
Respiratory/Thoracic	3		
Other	2		

### Interviews

Written informed consent was obtained from patients and caregivers prior to interviewing. Individual interviews were conducted with patients and caregivers separately in all but one case. A semi-structured interview format was used for flexibility, but with some focus to examine the phenomenon of interest. The present article reports on pain management as part of a larger investigation, where questions regarding pain perceptions and experiences were also included [40]. For examples of patient and family caregiver interview questions regarding pain management refer to the subsection below. The interviews were conducted by the first author (CM) and English and French-speaking graduate nurses with experience in palliative and/or cancer care and qualitative interviewing. Following each interview field notes were taken to capture contextual information such as the setting and participants’ reactions (e.g. body language and tone), as well as the interviewers’ preliminary insights.

#### ***Patient***

○ How do you typically respond when you are in pain?

○ What do you do to show your caregiver that you have pain?

  *Prompts/follow up questions:*

  Do you spontaneously report your pain or wait to be asked by your caregiver?

○ Are you able to communicate your pain to your family caregiver?

○ How does your family caregiver assess your cancer pain?

  *Prompts/follow up questions:*

  In what ways?

○ How do you deal with cancer pain?

  *Prompts/follow up questions:*

  What helps to control the cancer pain?

○ What are some of the challenges you face with managing cancer pain at home?

○ How do you see your role in managing your pain?

  *Prompts/follow up questions:*

  What is your family caregiver’s role in managing your cancer pain?

○ Do you discuss these roles?

○ Do you have any concerns about how your cancer pain is/will be managed (currently, in the future)?

#### ***Caregiver***

○ How does _________ typically respond when he/she has pain?

○ Do you feel that your _______ is able to communicate his/her cancer pain to you?

○ How do you assess your _________ cancer pain?

  *Prompts/follow up questions:*

  What types of information do you take into consideration when you assess his/her pain?

  What helps in assessing his/her pain?

  What hinders pain assessment?

○ What do you do when _________ he/she is in pain?

○ How do you see your role in managing your _______cancer pain?

  *Prompts/follow up questions:*

  Is managing your ______ pain a role you want to play?

  Do you feel adequately prepared to help in the management of his/her cancer pain?

○ What are some of the challenges you face with managing cancer pain at home?

○ Do you have any concerns about how your _______ cancer pain is/will be managed (currently, in the future)?

### Data analysis

All interviews were audiotaped and transcribed. The transcripts were compared against the audio-recordings for accuracy by the first author (CM). The interviewers’ field notes were also added to each transcription to provide greater contextual information and enhance interpretation. NVivo 10, a qualitative analysis software program was used to organize the data for analysis. Data analysis was conducted by the first (CM) and third (AD) authors. All individual interviews were read in their entirety several times to become familiar with the data. Questions and responses specific to pain management were separated from those exploring pain perceptions since this was the focus of the inquiry. The manifest or surface level content was initially coded to identify and describe the types of assessment, cues to pain, and expressions of pain explicit in participants’ responses. A thematic analysis was conducted to gain a deeper understanding of the underlying latent content of the responses within their context and to capture the richness of the phenomenon, [41]. Each transcript was read and reread in its entirety and across cases (patient-caregiver dyads) to capture the latent content. During this process subthemes were developed, modified, and combined to form themes. A theme, as Boyatzis [46] identifies is "a pattern in the information that at minimum describes and organises the possible observations and at maximum interprets aspects of the phenomenon" (p. 161). Continuous reference was made to the raw data to ensure the analysis represented participants’ meanings and experiences. An audit trail of decisions was kept throughout the process of analyses. To further enhance trustworthiness [42], a criterion relevant for quality in qualitative research, once all the transcripts had been coded and themes and subthemes identified by the first author (CM), the third author (AD) analysed the data independently. Consensus was reached on the coding, subthemes, and themes prior to review by the research team (CM, TH, ML, AD), which comprised of individuals with varied relevant backgrounds (nursing, psychology, gerontology, and oncology) and experience in qualitative research methods.

## Results

**Figure 1:**
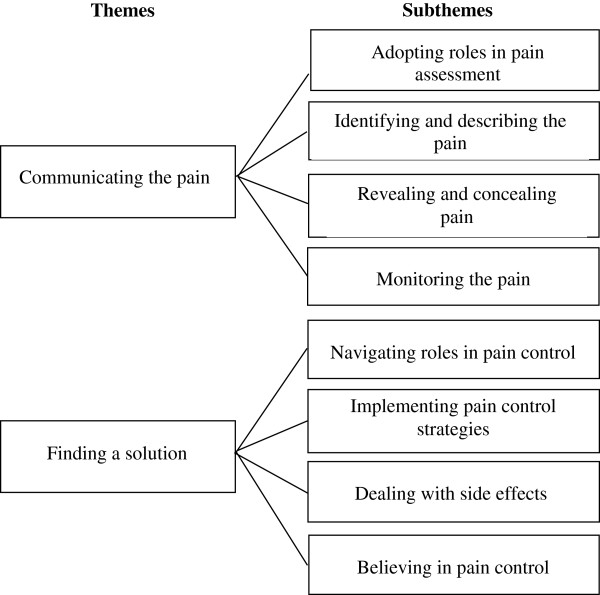
Themes and subthemes representing patients’ and family caregivers’ roles and perceptions in cancer pain management.

The focus of the inquiry was on patients’ and caregivers’ management of pain. From an analysis of the data two overriding themes were identified; *"Communicating the pain"* represented pain assessment, while *"Finding a solution"* reflected pain treatment. Figure 1 contains the focus of the inquiry, themes, and subthemes. The dyadic nature of the inquiry was best illustrated by reporting the findings from patients and caregivers together.

### Communicating the pain

*"Communicating the pain"* encompassed four subthemes. *"Adopting roles in pain assessment"* reflected patients’ and caregivers’ roles in pain assessment, while *"Identifying and describing the pain"* incorporated participants’ descriptions of pain and the challenges they experienced. Patients’ verbal and non-verbal expression of pain were identified in the subtheme *"Revealing and concealing pain",* whereas "*Monitoring the pain"* captured caregivers’ perspectives on pain assessment.

#### Adopting roles in pain assessment

Patients adopted the lead role in pain assessment; reflecting the subjective nature of the cancer pain experience. In contrast, caregivers had more limited roles and awareness of patients’ pain, particularly when patients were self-managing pain, or did not live with the caregiver. Talking about his wife one patient commented,

"*Well, I mean basically most of the time, she won’t even know I’m in pain. That I am taking the medication that I am on. I take it myself. I am self-medicating" (P 15).*

Further restricting their role was the finding that patients were not always forthcoming with information regarding their pain, and caregivers were sometimes not included in discussions with health care professionals visiting the home. Even with this limited role, however, caregivers spoke of being watchful and observing patients for signs of pain, and checking patients’ notes and medication records to see how many analgesics had been taken. They also supported patients by attending health care appointments outside of the home. The following comment best describes these roles for the most part,

"I guess …as part of the team. He’s, the number one player, always. Then there are the doctors who help him. I support him. Like with speaking up when he is really in pain and he doesn’t discuss it with the doctors" (FCG 15).

Declining patient health and functioning meant a corresponding increase in caregivers’ roles in controlling pain; knowledge of patients’ pain became necessary with this active role. As a consequence, caregivers became more involved in discussions with health care professionals, alongside the patient. Even so, clinicians’ reliance on patients’ self-report of pain meant that patients retained a central role. The presence of cognitive impairment was the only factor that impacted patients’ ability to comprehensively report their pain. In these instances, caregivers’ assessments supplemented the information provided by patients to health care professionals. Regardless of patients’ and caregivers’ typical roles and responsibilities, escalating uncontrollable pain diminished patients’ levels of functioning, and caregivers became key in initiating contact with health care professionals.

### Identifying and describing the pain

Most patients were able to provide information on the characteristics of their pain in terms of the physical sensation (e.g., *"stabbing", "burning", "sharp"),* frequency, location, and temporal characteristics (e.g., increasing, intermittent). They were also able to identify when pain was most likely to occur (e.g., at night, in the morning) and factors likely to exacerbate it (e.g., moving). Patients with cognitive impairment and their caregivers were more limited in their descriptions (i.e., location, severity). As the pain scale from the ESAS was used clinically, most patients provided a number indicating the intensity of their pain, which was useful for describing the intensity of the pain and monitoring the effectiveness of strategies to control it, as the following quote from a daughter caregiver illustrates,

"If she says yes, I have pain, then we will use the scale [ESAS pain scale]. We ask is it stronger than before? …. If she cannot describe the pain, I will ask her from 0 to 10, how much is the pain?" (FCG 3).

Reference was frequently made by patients to pain in various locations around the body, which made it difficult for patients and caregivers to identify the origin of the pain. Painful comorbidities (e.g., arthritis, diabetic neuropathy, hernia and lupus) and symptoms (e.g., constipation, swelling, muscular pains and stomach problems), added another level of complexity to the assessment, as the following comment illustrates, *"…you know it’s hard sometimes to differentiate what’s holding her back. Is it the pain or is it the effects of the chemo…" (FCG 5).* Albeit, pain that was localized to the primary cancer site, areas of metastases (i.e., headaches, back pain), and distinctive pains such as *"pin and needles"* from diabetic neuropathy, were easier to identify. Interestingly, some patients did not identify pain as such; instead it was referred to as *"discomfort", "cramp"* or an *"ache".* Expectations regarding suffering with advanced cancer and death became a marker for current pain, as one patient remarked, *"I can’t say that I have suffered terribly. I don’t know what is down the road but I understand bone cancer can become very painful" (P 5).* At the same time, increasing pain or new pains brought concerns given the association with advancing disease and death.

### Revealing and concealing pain

Most patients stated that they were open and verbally communicated their pain to caregivers. However, concerns about bothering caregivers meant that often patients would avoid telling caregivers until pain became intense and difficult to manage, as the following response illustrates,

"Yes I tell her but I don’t want to stress her out any more about it …. I wait until she asks you know. She is busy with her own life and her own things. I don’t want her to have to worry about me you know" (P 7).

There was also reluctance to communicate pain in couples who were both dealing with longstanding health issues (e.g., cancer and arthritis), to protect one another from bearing additional pain. Describing the communication of pain in his relationship, a patient stated *"its [pain] just an accepted part of our relationship" (P 15).* He went on to add that dealing with pain continually meant, *"We got to the point where we communicate only about our pain, only when it is was so overwhelming" (P 15).*

Pain was expressed non-verbally and verbally. Vocalisations of different sounds indicative of pain were sometimes referred to and comprised of moaning, whimpering, and interjections such as *"ouch".* Non-verbally, facial expressions such as grimacing or wincing and behaviours aimed at alleviating the pain, such as holding, rubbing or massaging the painful area, and taking analgesics were described by patients. A daughter caregiver described her mother’s reaction to pain in the following way,

"The traits of her face.. they are not the same traits. The way she speaks too. The voice will also weaken when she has pain. If she suffers, the tone of her voice drops. She will not speak clearly. Then when she has really bad pain the tone of her voice drops much more" (FCG 3).

Living with pain affected all aspects of patients’ lives to some degree and these effects on functioning were often used to illustrate the intensity of the pain, as one patient described, "*Well it started out at Christmas where it [the pain] was crippling me. I couldn’t even walk and every step was an eternity with the pain. I can’t describe how terrible it was" (P 2).*

### Monitoring the pain

Although caregivers’ mentioned discussing the patients’ pain, illness, and its management with health care professionals, learning how to assess pain was, for the most part, a case of figuring it out. As one caregiver remarked, *"I just. I just … I JUST DO" (FCG 15).* Verbal interaction with patients was the main approach to assess pain. Often non-verbal indicators were used to initiate these interactions, or to corroborate the pain communicated verbally. Caregivers’ knowledge of the patient was integral in making their assessment. Caregivers’ familiarity with the patient and constant contact meant that they had baseline knowledge from which to observe for changes, *"I feel that I have an advantage because I know him. I know his background. You know. I know how he looks, how he compares to today, compared to yesterday, or a year ago" (FCG 15).*

This knowledge and experience meant that caregivers became accustomed to how patients responded to pain. For example, caregivers were often aware of patients’ reticence to communicate their pain,

"She says it when it is worse. That’s how we know. When she says: "I have pain", it is when it is worse. I know that she has pain and that she will not say it each time that she has pain" (FCG 13).

The communication of pain in these cases became an indicator that the pain was overwhelming. Given patients’ reluctance, caregivers relied on non-verbal indicators of pain that corresponded with patients’ pain expressions and behaviours. Visual pain indicators such as restricted functioning were more apparent when pain was intense, and not only provided information on the presence and intensity of pain, but also the location*.* This meant that some types of pain were easier to identify than others,

"His arthritis pain, I can’t tell because unless he tells me about it, I don’t see it on him. You know? For the mouth pain, I can tell whether he was eating or not. You know, or whether he is drinking or not" (FCG 15).

At the same time, reduced functioning was not always a reliable indicator of pain because fatigue and weakness also affected patients’ functional level,

"Well, I don’t know much about pain, his pain because he has always had it. You know … the only indication is when he sleeps more but now he is sleeping more because he is tired and from the cancer treatment" (FCG 15).

For some, especially older partner caregivers, their own experiences with illness (i.e., advanced cancer, arthritis, diabetes) and associated pain, helped in understanding some types of pain*.* However, not all caregivers had this knowledge, experience, or contact with patients, which made pain assessment difficult without the verbal communication of pain. Speaking about her mother, a daughter caregiver acknowledged, "*"I know visually when she needs a pain pill. I might see her go get one but to watch her moving or listen to her talking I don’t know unless she says I need one" (FCG 5).*

### Finding a solution

*"Finding a solution"* consisted of four subthemes. In the subtheme "*Navigating roles in pain control",* patients’ and caregivers’ roles in managing patients’ pain were identified*. "Implementing pain control strategies"* included various pharmacological and non-pharmacological approaches to pain relief, while unwanted side effects from pharmacological approaches were identified in *"Dealing with side effects". "Believing in pain control"* captured participants’ beliefs and expectations regarding cancer pain and its treatment.

### Navigating roles in pain control

Participants assumed various roles in pain treatment. There was a strong desire from patients to maintain independence in managing their pain,

"He is good with his medication…He tends to be very independent that way. He likes to take it himself. He does not consult me or want me to administer it for him" (FCG 15).

In cases where patients were self-managing with pain medications caregivers’ played a more supportive role with responsibility for attending appointments and obtaining medications. Still, functional and sensory losses associated with aging, co-morbidities, symptoms, and side effects from treatments contributed in making self-management difficult. Even in instances where patients were largely responsible for deciding on the pain treatment approach, caregivers living with patients were involved in pain treatment decisions and assisted with the administration of non-pharmacological approaches (e.g., massage, mobilizing, changing position, and applying heat or cold) and pharmacological approaches to pain relief (e.g., pushing the button on the medication pump, assisting the patient to take medications). Of particular note were impairments in patients’ cognitive functioning, often referred to as *"forgetfulness",* which would necessitate caregiver intervention in terms of reminding the patient to take medications and monitoring analgesic use. This raised concerns when patients were self-medicating. A partner caregiver expressed his apprehension,

"Her memory is not as good as it was in the short term. She knows it because sometimes she says "I don’t know if I took it [analgesic]". That is dangerous, so I see if she took it first, I ask her if she took it, then … I talk with her "would you like to take this or that pill, this painkiller or another?"(FCG 17).

This created frustration and distress for caregivers who witnessed unnecessary suffering as a result of patients forgetting to take medications for pain. Changes in cognitive functioning also blurred patients’ and caregivers’ roles, which were not easily negotiated and became a source of conflict for some,

"Well, I cannot take him in hand anyway. Sometimes when he forgets to take his drugs, when I remind him, he does not like that.. it’s perhaps all that belongs to him now. For that reason, I cannot tell him" (FCG 9).

Increasing functional impairment brought with it greater caregiver responsibility. For some the responsibility was extensive including deciding on the most appropriate pain treatment options, administering non-pharmacological interventions, and complex prescribed pharmacological regimes, and controlling side effects. As one caregiver described,

"I tried to take him down to 1515 but it [analgesia] was much too low. He started having withdrawal; he’s having a withdrawal breakthrough now. His pain is starting to breakthrough a bit now even with the 1815. I have to go back up. I manage the going up and down. I manage the constipation as much as I can. I gave him three enemas the other day because sometimes he needs enemas to relieve himself. He gets so constipated" (FCG 2).

### Implementing pain control strategies

Pain control strategies could be divided into pharmacological and non-pharmacological approaches. Pharmacological approaches were most often mentioned and consisted of long acting medications for cancer pain, medications for breakthrough pain, and adjuvant medications (e.g., anticonvulsants and antidepressants). Several patients were prescribed an opioid pain medicine that was administered by mouth, a transdermal patch, or morphine pump. Participants also mentioned other analgesics used for non-cancer pain (e.g. arthritis) and comorbidities. Although pharmacological approaches were the predominant response, patients were often reluctant to use them, especially for acute breakthrough pain. Instead, patients would prefer to use a "*wait and see"* approach where they would wait to see if the pain could be tolerated, or would resolve without pharmacological intervention. Describing her mother’s response to pain, a caregiver responded,

"Mom has a very high pain tolerance and she is of the mind that I don’t take anything until I really need it. So it took a while to learn that in this instance you don’t wait until you really need it because then you need too much or it takes too long" (FCG 5).

Caregivers on the other hand were more apt to use pharmacological approaches and would encourage patients to do the same. Non-pharmacological interventions, as illustrated in Table 2, were preferred by patients, in particular: lying down, sleeping, changing position, and not moving. Distraction was also used by some, although it had limited effectiveness when the pain was intense*.* Particular strategies were chosen to alleviate different types of pain. If the pain was attributed to arthritis, or soreness due to remaining in one position for too long, then massage and repositioning were used. Elevating the patient’s legs helped with pain due to swollen legs and circulatory conditions, while heat eased muscular type pains.

**Table 2:** Non-pharmacological approaches to pain treatment

**Pain treatment approach**	**Illustrative responses**
Moving/changing position	*"Well I will get up and move around and if it is gone within a few minutes" (P 2).*
	*"I would have to lift both his legs at one time raised them up to put him in prop him up with the pillows it was a bad time you are rubbing things on it hoping that rubbing this medication will help it and he was only on anti-inflammatory than he wasn’t on painkillers" (FCG 10).*
	*"What I do find, sometimes, if I have been sitting for a while … when I first get up .. it is like a cramp. Then after I walk around for a few minutes then it goes off" (P 13).*
	*"Soon as I change of position, very often, it goes. I just have to lie down, stretch out then it goes. Just to change position helps me a lot" (P 17).*
Not moving/resting	*"It can get very sore but I can go and sit down and in five minutes it is fine" (P 5).*
	*"There are medications but before they take effect.. I try to sit as still as I can, or go lay down.. just resting helps" (P 12).*
Distraction	*"I try to go to seek a distraction. Now I have something to read" (P 3).*
	*"While we are talking… I forget everything. Even if I have pain. I forget" (P 6).*
	*"I try to keep my mind busy.. sometimes I read .. or I watch TV (P 18).*
	*"Listening to music… working on the computer. I’m quite good at it. And.. categorizing the thousands and thousands of slides and pictures I have taken over the years…That is the way I cope with my pain" (P 15).*
	*Recently what helped me a lot me. I began again to knit. I am a big, big knitter, I made absolutely extraordinary things… and then, so that occupies me" (P 9).*
Talking/being with others	*"When I have somebody with me, let’s say that the pain is shared. Everything is shared for me and I am not sick. I see myself well when I am with someone" (P 3).*
	*"I think that, that I am a painkiller for her. Because sometime she panics and then to see me there, not in panic, it helps her" (FCG 17).*
Heat/cold/massage	*"When she has pain in her leg, her right leg I think, we can apply ice for the pains she has" (FCG 3).*
	*"I get relief if I put hot water on it and when I am showering" (P 6).*
	*"Rubbing things on it sometimes helps" (P 18).*
	*"I will rub it a bit, but as soon as I move, it goes away" (P 12).*
Equipment	*"We got a hospital bed. That has been his Godsend.. That mattress helped his pain level .. it has a nice thick foam" (FCG 2).*
	*"That chair has been great for her because it is automatic she can lift her legs up, get the circulation up and it helps with the pain" (FCG 4).*
Humour/outlook	*"Well I try to think positive.. that sometimes helps" (P 12).*
	*"My sense of humour always … laughing or watching other people laugh. Seems to reduce the pain for some reason" (P 15).*
Faith	*"What helps me? My faith" (P 16).*
	*"If I can’t fall asleep, I have the time to do some prayers and finally the medication is going to take effect" (P 9).*

### Dealing with side effects

A common concern associated with pharmacological approaches was unwanted side effects. Participants spoke of patients being *"dopey", "getting mixed up",* and *"forgetful",* although they were unsure whether it was a consequence of aging, the disease process, or the medications. Some side effects paralleled the cancer- related symptoms of fatigue and weakness making it difficult to differentiate between the two symptoms. Medication side-effects added to functional limitations, for example, patients with pre-existing mobility problems became more unsteady, which reduced usual activities and became a safety concern. Certain medications also prevented patients from engaging in activities such as driving and using equipment. Morphine pumps were also seen as an inconvenience. Indeed, some patients showed a preference for pain over the side effects, "*Cause he (caregiver) keeps saying "take your medication. Take your pain killer. Stop the pain" But, I would rather have the pain than be constipated" (P 13).*

### Believing in pain control

The majority of patients believed that pain was inevitable and could not be entirely controlled so there was "acceptance" of some level of pain. Difficulties gaining control over the pain further contributed towards this. As a result, the goal of pain treatment for many patients was to reduce the pain to a tolerable level, while minimizing unwanted medication side effects. There was some reticence toward pharmacological approaches. Patients’ expressed concerns regarding opioids because of fears about tolerance and addiction; which sometimes led them to delay, or omit taking medications.

"I don’t want to take the extra morphine but sometimes I may have to. [Interviewer] What would be some of the reasons you would not want to take your medication if you’re having pain? [Patient] Not to get too addicted to it" (P 2).

Still a few patients who were initially hesitant to take medications became more confident in taking analgesics on a regular basis, when they gained a better understanding of the medications and their effectiveness,

"I realized from trying to not take pills a few months ago that in a sense .. I was more or less doing myself more harm than good by trying to be this little brave old lady that could do without the pill. And after that I realized you know, like if you speak to me and put the pros and cons out. I will try to get the picture" (P 8).

Caregivers often shared the belief that pain was inevitable with advanced cancer and old age, but were more varied in their beliefs regarding pain control and the goals of pain management; though a few expressed unease about the number of medications patients were taking given their age. Revealing her belief that pain was inevitable, a daughter caregiver remarked,

"*My concern is how long can someone like her be on this many painkillers… she was on them for almost a year. It’s just that it’s such a strong medication, and it’s not good for you, like you take painkillers because you are in pain, then the pain goes away, but in her case it will never go away" (FCG 4).*

Other caregivers believed that medications should control the pain. However, achieving control was characterised as a *"battle"* that took time, since changes in patients’ pain over the illness trajectory meant ongoing modifications to medications. Once pain control was achieved, caregivers were more confident and less concerned about the use of pharmacological approaches. Comparing the goals of pain treatment at the dyadic level revealed the shared overall goal of avoiding patient suffering, and the preference for home care, however, differences in patients’ and caregivers’ expectations and beliefs regarding pain, and its treatment indicated that the specific pain treatment goals were not always congruent. Comparing dyadic responses revealed that patients were more apt to tolerate pain by reducing or coming off medications, specifically opioids, if it meant a reduction in unwanted side effects and they could remain at home. Caregivers, on the other hand, favoured the continued use of analgesics including opioids to eliminate the pain and avoid in-patient care.

## Discussion

The purpose of this study was to explore and describe the roles and perspectives of older patients with advanced cancer and their family caregivers in pain management in the home. The findings, similar to those from other studies [31-33], illustrates the complexities associated with pain in advanced cancer and challenges facing patients and caregivers. The emphasis on caregiving dyads (patients and caregivers) rather than on the separate experiences of patients and caregivers gives credence to the interactional process of caregiving, and varying roles and responsibilities they assume in assessing and treating pain. Distinct from other studies [23,31-33,35], the focus on older patients provides insights into their unique perspectives and experiences, and those of their caregivers.

Participants described their roles and responsibilities as evolving over time in concert with changes in patients’ functioning and needs. In some instances, patients were managing their pain with minimal assistance from caregivers. Though there has been an emphasis in the literature on caregivers’ experiences in managing patients’ pain, the findings, similar to others, indicate that even at the end of life patients are able to maintain an active role in managing their health care needs [47]. Patients’ engagement in their care is important because it ensures that their preferences are included in decisions regarding treatments and goals of care. Moreover, active involvement can be a coping strategy, which can alleviate distress and contribute to the acceptance of life-limiting disease [48]. Self-management of pain, therefore, should be encouraged, whenever possible, and strategies in place to facilitate this process and accommodate any functional or sensory deficits as a result of aging and disease [49].

At the same time, it is important to be mindful that progressive disease and declining functioning may compromise the ability of patients to self-manage. Although we did not include a formal assessment of cognitive functioning, it became evident during the interviews and through discussion with caregivers that some patients were manifesting indicators of a mild level of cognitive impairment. This type of impairment was a serious concern for the caregivers of patients who were self-medicating with pain relievers; overmedication was a safety issue, whereas, under medication compromised pain control. Cognitive impairment is a significant problem facing older patients with cancer as it not uncommon in advanced cancer due to the disease, treatments, and psychological sequelae [50]. Cancer pain and opioids used to relieve cancer pain can also produce cognitive impairment [50]. Importantly, older people are at increased risk of cognitive impairments due to age-related physiological changes, co-morbidities, and the presence of pre-existing cognitive decline [10]. These points taken together emphasize the need for comprehensive ongoing assessment of patients’ functioning; including a determination of their cognitive status, competency, and role in managing pain as changes occur over the disease trajectory. The association between psychiatric disorders and cognitive difficulties, together with the high prevalence of psychiatric conditions in advanced cancer and cancer pain [50], points to the need to include an assessment of psychiatric functioning, and the implementation of interventions to alleviate psychological and existential distress. Caregivers could also be instructed to be watchful for indicators of changes in patients’ functioning that could compromise patients’ abilities to manage pain. Crucially, formal supports need to be in place to ensure ongoing contact and monitoring of those patients living alone that have less contact with their caregivers. These patients may be at particular risk for suboptimal pain management as functional impairments may limit their ability to assess, select, and implement strategies to control pain.

Although patients’ and caregivers’ roles were generally described as cooperative and supportive, this was not always an easy alliance. There was reluctance by some patients to relinquish roles, preferring instead to maintain independence despite requiring assistance. These findings may represent patients’ attempts to maintain a level of control over the situation as they deal with losses associated with role changes, cancer, pain, and dying. Certainly, patients’ reluctance to disclose information regarding their pain constrained caregivers’ awareness of pain and its treatment and, to some extent, caregivers’ involvement in managing pain. Patient’s rationale for concealing pain from others was to avoid burdening others; a finding observed in other studies [26,27]. The sense of burdening others, or self-perceived burden, however, may serve not only to minimize the burden of responsibility on others, but protect the self from the sense of increasing dependence and changes in established roles and responsibilities [26]. These factors can affect the care provided, help sought, and receptiveness to help that is offered. Therefore, cancer pain and its meaning needs be assessed within the broader context of patients’ functioning and the caregiving relationship, as patients and caregivers adjust to the changes brought about by life-limiting disease and aging. The comprehensive assessment of pain is clinically relevant given the multidimensional nature of pain, and the associations between psychological distress and the experience of pain [20,21]. By framing pain assessment in this holistic manner, those at risk for maladaptive coping and conflict within the caregiving relationship can be identified. Interventions to facilitate the negotiation of roles and open communication of needs, including pain communication, between caregivers and patients can be implemented. In some instances, specialist help (e.g., clinical psychologist or social worker) may be required to assist patients and caregivers adjust to their circumstances.

All patients, including those who showed signs of mild cognitive impairment, were able to provide an assessment of their pain. This observation corroborates the well-established finding that patients with mild and moderate cognitive impairment are able to provide a valid self-report of pain [51]. These findings support practice recommendations to use patients’ self-report of pain whenever possible [52]. Also supporting extant practice guidelines, we found that the Edmonton Symptom Assessment Scale (ESAS) was useful for communicating and monitoring pain intensity and the effectiveness of interventions to control pain [44]. Beyond pain intensity, however, there were inherent difficulties in assessing pain and identifying the sources of pain due to the multiple physiological causes of cancer pain and the presence of pain and other symptoms that were cancer and non-cancer related. Our findings suggest the need for training directed toward helping patients and caregivers better identify and assess the type(s) of pain that older patients can experience with advanced cancer.

In our study, verbal communication between the patient and caregiver was the most significant pain cue; however, similar to findings from other studies [26,27], patients were reluctance to communicate their pain. Instead, caregivers relied on various non-verbal cues to the presence of pain. These cues fitted with characteristic manifestations of pain and may be salient indicators of pain, especially in the absence of patients’ self-reported pain [53]. However, it is important to be alert to the fact that some cues did not adequately discriminate pain from other symptoms; decreased functioning, for instance, could signify symptoms such as fatigue, weakness, or side effects from treatment rather than pain. Moreover, atypical expressions of pain such as confusion, depression, and withdrawal can occur [10] in the aged; thus emphasising the need for patients and caregivers to be aware of indicators and types of pain. An advantage for older patients and caregivers observed in our study, was that their knowledge, experience, familiarity, and contact with one another was helpful in identifying familiar types of pain, and typical and unique pain behaviours. Research has demonstrated that nonverbal pain cues are most likely to be considered by caregivers who spend more time with the patient when compared to caregivers who spend less time [54]. Even though research has shown that caregivers’ assessments of patients’ pain does not completely mirror those of the patients [55], they are an important source of additional information on the patient’s pain experience. Furthermore, non-verbal cues to pain may be used to initiate verbal communication of pain with the patient; as seen in our study. This type of communication should be encouraged to validate caregivers’ inferences regarding patients’ pain with the patient, and to develop a joint understanding of changes in patients’ functioning as it occurs.

For these older patients and their caregivers, previous experiences with pain and symptoms from comorbidities meant that they were knowledgeable about various treatment options and what had worked for them in the past. There was some evidence that pain strategies were linked to different types of pain, particularly for pre-existing non-cancer chronic pain. Patients’ expressed a definite preference for non-pharmacological approaches (Table 2); a finding observed by others [56]. These approaches might be viewed as an alternative to pharmacological approaches by older people and reflect their reluctance to use medications. We found, like others [36,39], that there was some reluctance to take opioids because of fears about tolerance, addiction, and side effects. Side effects may be a real concern in the older population because of the potential for polypharmacology and adverse reactions with the presence of disease and reduced physiological functioning [10]. Nonetheless, age does not preclude the use of opioids prescribed on the basis of a comprehensive clinical assessment of the patient, and in conjunction with prophylactic pharmacological interventions to counteract side effects [52]. Monitoring patients’ responses to different pain treatment strategies can help to determine their individual effectiveness and to identify adverse reactions. Also, older patients’ preference for non-pharmacological approaches to pain relief [56] could be supported in combination with pharmacological approaches to maximize effectiveness and minimize side effects.

Importantly, the goals of pain management should not be based on misconceptions regarding the inevitability of pain, but informed by the effectiveness of interventions and patients’ preferences. Patients and caregivers in our study did not share the same goals when it came to pain management; even though the ultimate objective was to maintain care within the home. Thus the goal to be pain free cannot be assumed in older patients since many factors play into their decisions. Maintaining independence, for example, may be more important with a tolerable level of pain, than to be pain free and unable to function due to side effects. Understanding cancer pain within the broader context of patients’ and caregivers’ knowledge, beliefs, and experiences, is essential for appreciating their choices and pain management goals.

Consistent with others, our findings highlight that pain management for patients and caregivers is more than merely monitoring for pain and adhering to prescribed regimens [12,31-35]. Instead, patients and caregivers are jointly engaged on a daily basis in various roles that require knowledge of cancer pain and its management. Patients and caregivers need to be able to problem solve on treatment options taking into account patients’ preferences, treatment goals and side effects, and also have the skills to implement treatments [32]. Interventions have been shown to be effective in correcting misconceptions and increasing knowledge and self-efficacy [57]. However, few intervention studies have focused on older patients and their caregivers [58]. Yet, the complex care needs of an older patient population and those of their caregivers make it imperative that interventions be directed toward helping them to deal with pain and other issues they encounter. Noteworthy in our study was the finding that many of the caregivers were themselves older and dealing with health issues; in one case both the patient and caregiver had advanced cancer and pain. The prevalence of chronic disease in old age makes this more probable in some relationships (i.e. partner and sibling) and is a factor that needs to be considered by health care professionals in the provision of care. Consideration also has to be given to the type, amount, and presentation of information, since symptoms and age-related changes (e.g., sensory loss and declining memory) limit the processing of information [59]. Efforts, therefore, need to be directed toward generating and translating knowledge into practice, taking into account the particular needs and preferences of older patients with cancer pain and their caregivers.

### Limitations

The focus of our study was on older patients and caregivers. However, it is critical to acknowledge that barriers to optimal pain management occur at the system- and health care professional-levels, and that these barriers likely intersect to affect pain management at the individual patient- and caregiver-level [60]. Attention needs to be given to addressing barriers at all levels to meet the needs of patients with cancer pain and their caregivers. It is also important to recognise that the older population is heterogeneous and although comorbidity, non-cancer chronic pain, and cognitive impairment are more prevalent in old age and can make pain management more challenging, these are not an inevitable part of aging; not all older patients with advanced cancer and their caregivers will encounter these issues. Also, the focus was on the dyadic perspective from patients and their primary caregivers; however, care may be provided by more than one caregiver. Future research would benefit by exploring the dynamic interplay, roles, and perspectives of multiple caregivers who provide care to one family member. Furthermore, despite efforts to sample for different caregiving relationships, caregivers were mainly partners of the patient. Our findings indicate some differences between caregivers residing with the patient (mainly partners) and those not residing with the patient (adult children, friends). In light of an increasing number of caregivers who reside at a geographic distance from the patient, this would be a fruitful line of inquiry to pursue.

## Conclusion

Our findings support other studies in identifying knowledge and attitudinal barriers to pain control, while adding to the literature by highlighting practical and relational barriers faced by older patients and their caregivers. Health care professionals can do much to address the barriers identified by: correcting misconceptions regarding cancer pain and its management, facilitating the communication of pain within dyads, and ensuring that patients and family caregivers have the necessary knowledge, skills and ability to assess and implement the most appropriate pain treatment strategy based on patients’ preferences. This support needs to be individually tailored to meet the ongoing needs of both members of the dyad so that shared goals of pain management are accomplished.

## Abbreviations

PPS: Palliative performance scale; ESAS: Edmonton symptom assessment scale; SD: Standard deviation.

## Competing interests

The authors declare that they have no competing interests.

## Authors’ contributions

CM, TH and ML designed the study and developed the methodology; CM collected the data; CM and AD performed the analyses and TH and ML verified the analyses; CM and AD wrote the manuscript and TH and ML critically reviewed it. All authors read and approved the final manuscript. 

## Pre-publication history

The pre-publication history for this paper can be accessed here:

http://www.biomedcentral.com/1472-684X/13/39/prepub
